# Shear wave elastography for differentiating parathyroid neoplasms with malignant diagnosis or uncertain malignant potential from parathyroid adenomas: initial experience

**DOI:** 10.1186/s40644-022-00503-0

**Published:** 2022-11-22

**Authors:** Ruifeng Liu, Luying Gao, Xinlong Shi, Liyuan Ma, Ou Wang, Weibo Xia, Ya Hu, Yu Xia, Yuxin Jiang

**Affiliations:** 1 Department of Ultrasound, Peking Union Medical College Hospital, Chinese Academy of Medical Sciences and Peking Union Medical College, Shuaifuyuan 1#, Dongcheng District, Beijing, 10073 China; 2 Department of Endocrinology, Peking Union Medical College Hospital, Chinese Academy of Medical Sciences and Peking Union Medical College, Shuaifuyuan 1#, Dongcheng District, Beijing, 100730 China; 3 Department of General Surgery, Peking Union Medical College Hospital, Chinese Academy of Medical Sciences and Peking Union Medical College, Shuaifuyuan 1#, Dongcheng District, Beijing, 100730 China

**Keywords:** Parathyroid carcinoma, Atypical parathyroid tumor, Ultrasonography, Shear wave elastography

## Abstract

**Objective:**

Parathyroid carcinoma (PC) and atypical parathyroid tumor (APT) are rare parathyroid disorders carrying the risk of recurrence of varying degrees. This study aims to explore the value of 2D-shear wave elastography (SWE) in the discrimination of PC/APT among suspicious parathyroid lesions.

**Methods and materials:**

In this prospective study, patients with primary hyperparathyroidism and suspicious parathyroid lesions on ultrasonography (US) were recruited. All the lesions were assessed by SWE before surgery. The velocity (m/s), Young’s modulus (Kpa), and elastogram of SWE were compared between pathologically proven parathyroid carcinoma or atypical parathyroid tumor (Group1) and parathyroid adenoma (Group2). All the SWE parameters were displayed at the setting of 50 or 70 kPa. Correlations between SWE and the lesion size as well as biochemical parameters were analyzed.

**Results:**

36 target lesions were enrolled for analysis. The mean shear wave velocity (SWV) between the two groups was 2.4 m/s vs 1.9 m/s, respectively, while the mean Young’s modulus was 11.1 kPa vs 18.2 kPa, respectively. The cut-off values are 2.35 m/s and 17.05 kPa correspondingly. The sensitivity and specificity of the selecting cut-off values were 0.56 vs 0.63 and 0.95 vs 1.0 (area under the curve [AUC]: 0.813 vs 0.852 [95% confidence interval (CI): 0.669–0.956 vs 0.720–0.983]; *p* <  0.001, *p* <  0.001; respectively). In contrast, the max SWV and Young’s modulus showed a better sensitivity of 0.75 and 0.81, respectively. The “colored lesion” and “stiff rim” patterns on the elastogram are more indicated in parathyroid carcinoma and atypical parathyroid tumor, whereas the negative elastogram prevails in parathyroid adenoma. The SWV and Young’s modulus of the parathyroid lesions were independent of the tumor size, but the max SWV and Young’s modulus slightly correlated with serum parathyroid hormone concentration (PTH) (r = 0.398, *p* = 0.016; r = 0.396, *p* = 0.017).

**Conclusions:**

2D-shear wave elastography plays a useful role in the preoperative assessment of parathyroid lesions with suspicious malignancy. The mean SWV and Young’s modulus are advised as the favored diagnostic parameter with the best AUC and excellent specificities, while the max SWV and Young’s modulus are more sensitive to distinguish the PC and APT compared with other parameters.

## Introduction

Primary hyperparathyroidism (PHPT) is a biochemical diagnosis characterized by an abnormally elevated level of serum parathyroid hormone, with hypercalcemia or normocalcemia [1]. The entities responsible for this disorder include most frequently encountered parathyroid adenoma (PA) and rarely appeared parathyroid carcinoma (PC) and atypical parathyroid tumor (APT) [2]. The preoperative distinction between parathyroid neoplasm subtypes is of significance since en-bloc resection is often warranted for those tumors with malignant potential [3]. In our experience, parathyroid tumors with malignant diagnosis or uncertain malignant potential confirmed by pathology are usually firm and adhere to surrounding tissues in operation. With the advent of shear wave elastography, an evolving ultrasound technique employing transverse waves to detect the stiffness of medium, it offers radiologists the chance to feel the hardness of parathyroid lesions before surgery [4]. Therefore, we aim to explore the role of SWE in predicting parathyroid tumors which may have poor biological behavior and risk of recurrence preoperatively.

## Material and methods

### Patients and clinical data

This research adhered to the Declaration of Helsinki and was approved by the Institutional Review Board (No: S-K1518). Written informed consent was obtained from each participant**.** Eligible patients diagnosed with primary hyperparathyroidism between July 2018 and July 2022 were enrolled in the cohort. All of them have parathyroid tumors with size> 1.5 cm on ultrasonography. In addition, the candidates must equip at least one of the following suspicious malignant features: (1) serum iPTH > 500 pg/mL or serum calcium> 2.9 mmol/L; (2) lesions with irregular shape or infiltrative border on ultrasonography; (3) newly-onset lesions after previous parathyroid surgery (suspicious of recurrent parathyroid carcinoma). Patients younger than 18 years old, without preoperative US imaging for target lesions confirmed by pathology or choosing the conservative management were excluded from this study. All of them received surgery after a multimodal neck ultrasound evaluation. Preoperative biochemical parameters including serum intact parathyroid hormone (iPTH, range 12-68 pg/mL), serum calcium (Ca, range 2.13–2.70 mmol/L), serum phosphorus (P, range 0.81–1.45 mmol/L), and alkaline phosphatase (ALP, range 35-100 IU/L) were analyzed. Symptoms in participants were classified into 3 categories by severity. Grade 0 referred to those without any complaint but discovered by physical examination. Grade 1 represented classical symptoms like palpable neck mass, kidney stone, fatigue, bone pain, and constipation. Grade 2 stood for symptoms requiring hospitalization, including but not limited to osteitis fibrosa cystica, fractures, pancreatitis, intractable vomiting, and hypercalcemic crisis. The definitive pathology diagnosis of parathyroid neoplasm phenotypes is based on the criteria set out by World Health Organization 2017. The patients were divided into two groups according to the pathology-Group1: parathyroid carcinoma or atypical parathyroid adenoma; Group2: parathyroid adenoma.

### Ultrasonography and SWE

All the patients were performed with B-mode ultrasound to locate the target lesions responsible for primary hyperparathyroidism while in the supine position. The candidate lesions were selected according to the concordant imaging results of the MIBI scan and US. Features detected by ultrasound including tumor sizes (anteroposterior by transverse by sagittal dimensions), diameters’ ratio (DR), echogenic texture, shape, boundary, calcification, and cyst change were recorded. DR was the ratio between the lesion’s maximum diameter and minimum diameter measured in all the dimensions. Echogenic texture findings comprise “homogeneous” and “heterogeneous”. Shape information contains “round or oval” and “irregular shape”. The boundary was described as “clear” or “infiltrative or blurred”. Three specialists with over 5 years of expertise in thyroid and parathyroid ultrasonography imaging defined the feature of each lesion through the best-of-three method.

**Fig. 1:**
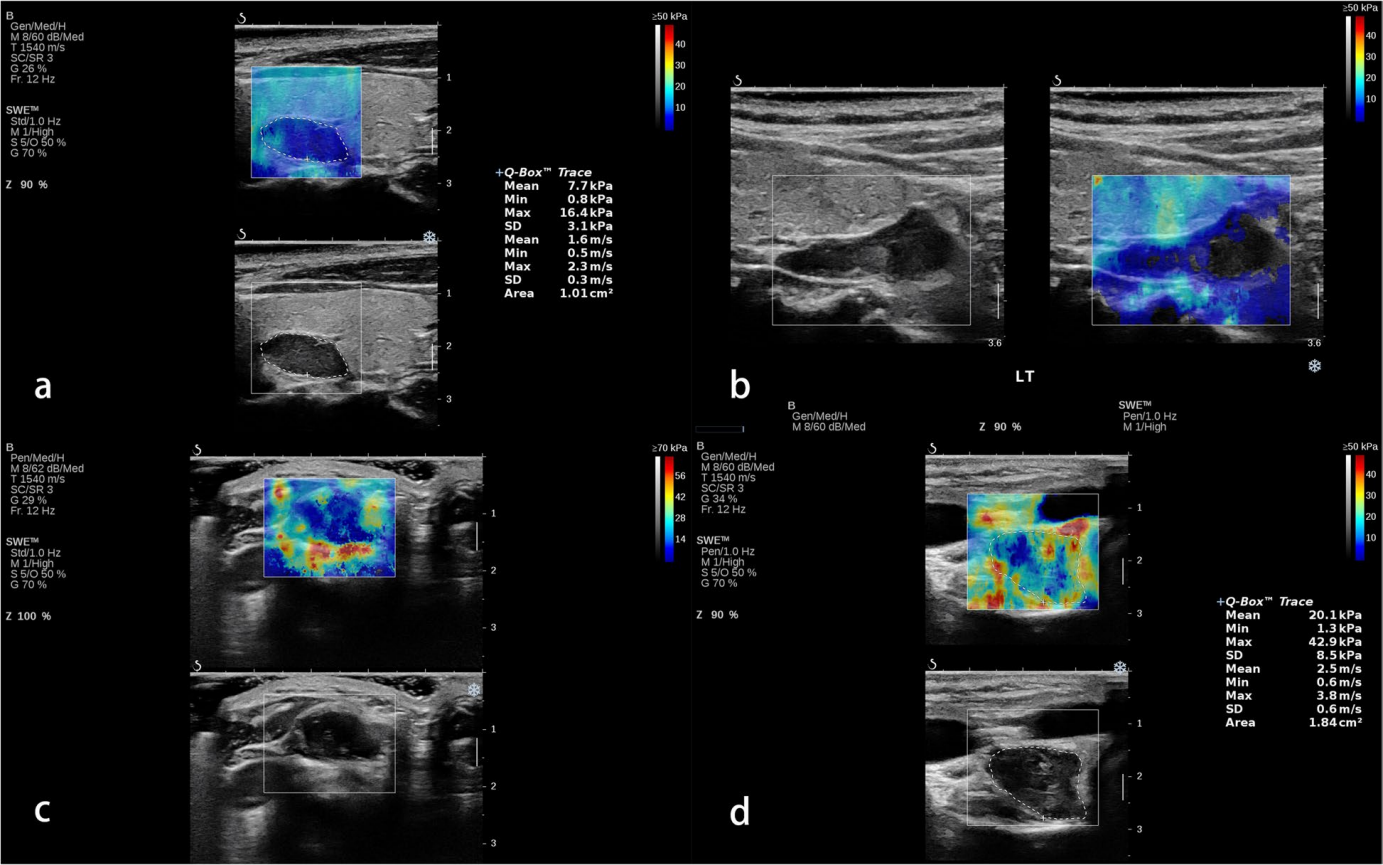
Four qualitative shear wave elastography patterns of parathyroid lesions. **a** The “negative” pattern was defined as no obvious color difference around and inside the lesion, displaying a homogeneously blue pattern; **b** The “void center” pattern was defined as the absence of color filling in the center of the lesion; **c** The “stiff rim” pattern was defined as increased stiffness (coded in orange or red) in the peritumoral region as compared with the stiffness in the surrounding soft tissues and the interior lesion tissues; **d** The “colored lesion” pattern was defined as the heterogeneously intralesional multicolor appearance

**Fig. 2:**
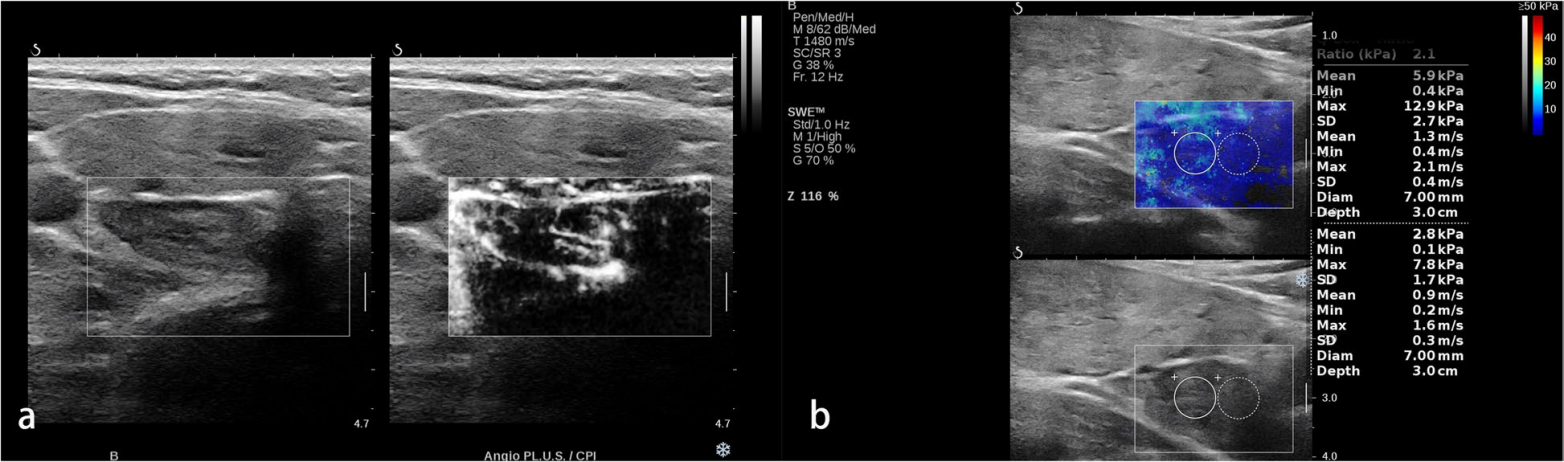
A pathologically confirmed parathyroid adenoma in a 69-year-old female. **a** The grayscale US and angio PL.U.S. vascularity detection technique (split-screen mode) showed a 2.8 cm hypoechoic solid lesion with rich blood flow located at the right neck. **b** The elastogram displayed at the setting of 50 kPa showed a negative pattern

**Fig. 3:**
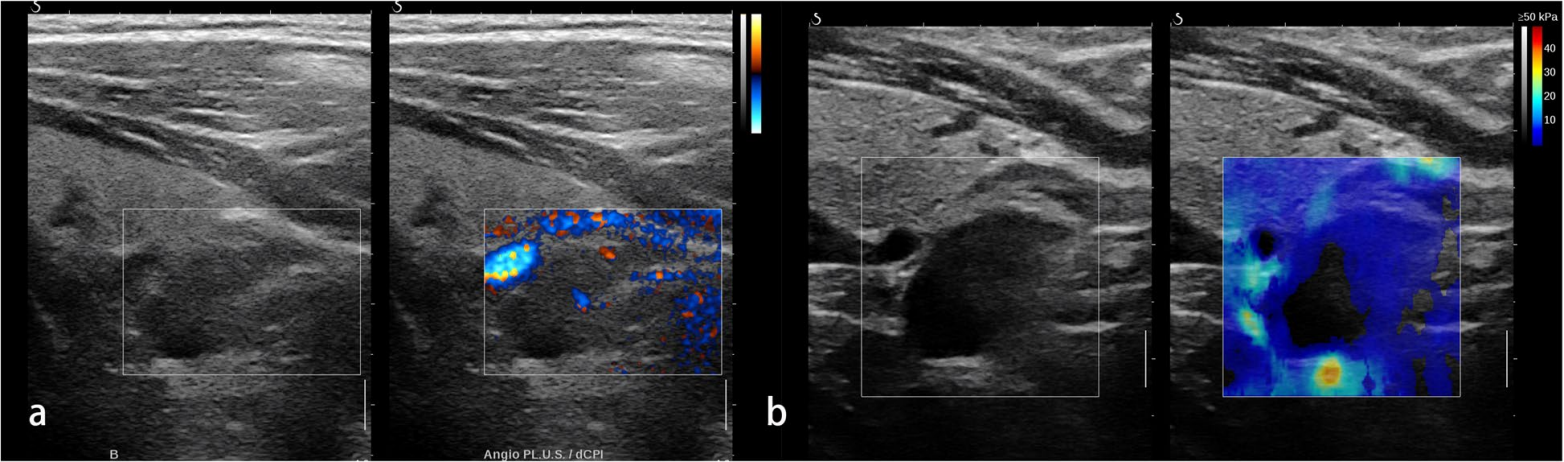
A pathologically confirmed parathyroid adenoma in a 53-year-old male. **a** The grayscale US and angio PL.U.S. vascularity detection technique (split-screen mode) showed a 2 cm hypoechoic solid lesion with blurred margin and intralesional hyperechoic stripe located at the right neck. **b** The elastogram displayed at the setting of 50 kPa showed a void center pattern

**Fig. 4:**
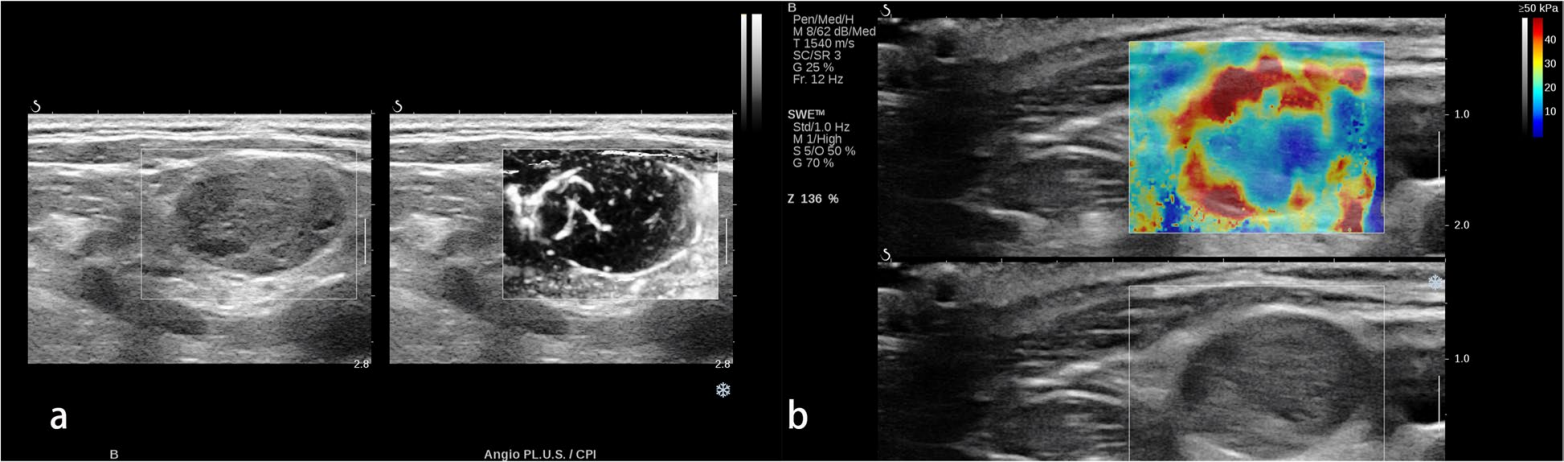
A pathologically confirmed parathyroid carcinoma in a 55-year-old female. **a** The grayscale US and angio PL.U.S. vascularity detection technique (split-screen mode) showed a 2 cm heterogeneous solid lesion with rich blood flow located at the right neck. **b** The elastogram displayed at the setting of 50 kPa showed a rim of stiffness pattern

**Fig. 5:**
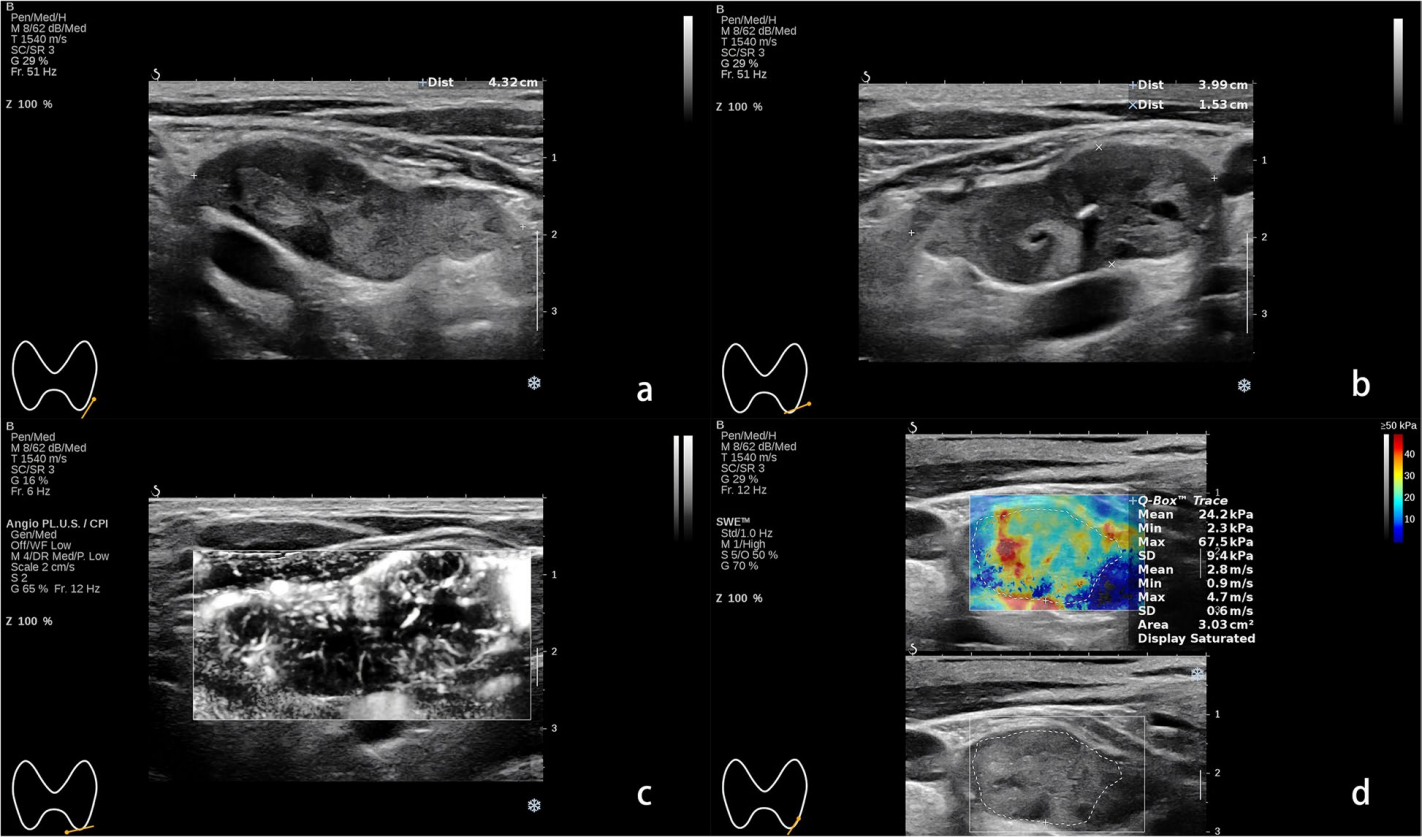
A pathologically confirmed atypical parathyroid tumor in a 55-year-old male. **a** The longitudinal view of grayscale US showed a solid lesion with irregular shape located at the left neck. **b** The transverse view of grayscale US showed the lesion was heterogeneous inside with calcification and liquid formation. **c** The angio PL.U.S. vascularity detection technique showed the rich and branched vascular distribution in the lesion. **b** The elastogram displayed at the setting of 50 kPa showed a colored lesion pattern

A senior sonographer with systemic training in SWE imaging performed the examination using an Aixplorer US system (SuperSonic Imagine S.A., Aix-en-Provence, France) with a linear probe (SL10–2). A guideline stated by WFUMB has been complied with during operation. The elasticity scale range was set in between 0 and 50 kPa in an evaluation firstly. For few cases whose max SWE parameters exceed the upper limit of 50 kPa, the scale of 0-70 kPa was applied. Region of interest (ROI) was traced conforming to the shape of lesions. As for large lesions with complex composition, ROI was placed inside the lesion and avoid the areas with cystic change and calcification. At least three measurements were conducted for each lesion. The median number of the mean SWE values in all the measurements for a single lesion was summarized for calculation. Parathyroid tumor stiffness was presented as shear wave velocity in meters per second and Young’s modulus in Kpa. For qualitative SWE pattern classification, the color-coded SWE elastograms were divided into four patterns: negative, stiff rim, void center, and colored lesion (Fig. 1). The “negative” pattern was defined as no obvious color difference around and inside the lesion, displaying a homogeneously blue pattern (Fig. 2); The “stiff rim” pattern was defined as increased stiffness (coded in orange or red) in the peritumoral region as compared with the stiffness in the surrounding soft tissues and the interior lesion tissues (Fig. 3); The “void center” pattern was defined as the absence of color filling in the center of the lesion (Fig. 4); The “colored lesion” pattern was defined as the intralesional heterogeneous multicolor appearance of a lesion (Fig. 5).

### Statistics

Data analysis was performed using IBM SPSS Statistics Version 25.0(Chicago, IL, USA) and STATA 15.0 (Stata Corporation College Station, TX, USA). Continuous variables are summarized as mean ± standard deviation and median ± interquartile range according to normality verified by Shapiro–Wilk test. Mann–Whitney U test was used for comparison of biochemical parameters. Age of patients, diameters on US and SWE variables were compared by independent samples Student’s t-test. Categorical variables including symptom grades were compared using the chi-square test or Fisher’s exact test and were shown as N and percent. Correlations between SWE and the lesion size as well as biochemical parameters were analyzed by the Spearman test. The receiver operating characteristic (ROC) curve was established and the area under the curve (AUC) was calculated to obtain a cutoff value for related variables of SWE. Sensitivity, specificity, positive predictive value (PPV), and negative predictive value (NPV) were calculated. A two-tailed *p* <  0.05 was the threshold for the statistical significance of the tests.

## Results

The demographic and clinical characteristics of eligible patients are reported in Table 1. Thirty-six lesions were enrolled in this study. Sixteen of the 36 lesions were PC(*n* = 8) and APT(n = 8), while 20 lesions were benign adenomas. No age or gender preponderance was observed between the two groups’ patients (*p* = 0.178). Serum PTH, Ca and ALP levels were markedly higher in Group1 compared with Group2 (*p* <  0.001, *p* = 0.005, and *p* <  0.003, respectively). Serum P level was significantly lower in Group1 relative to Group2 (*p* <  0.017). More severe symptoms presented in Group1 compared with Group2 (p <  0.001).

**Table 1:** Comparison of demographic, clinical and ultrasound parameters between parathyroid carcinoma/atypical parathyroid tumor and parathyroid adenoma

	parathyroid carcinoma/atypical parathyroid tumor (*n* = 16)	parathyroid adenoma (*n* = 20)	*p*-value
Mean age (years)	46.9 ± 12.7	52.9 ± 13.1	0.178
Gender (male/female)	6/10	8/12	0.878
Median PTH (pg/ml)	1384.4(441–1881)	241.3(108–529)	< 0.001
Median Ca (mMol/L)	3.39(2.96–3.61)	2.91(2.89–3.57)	0.005
Median P (mMol/L)	0.61(0.50–0.78)	0.82(0.50–0.94)	0.017
Median ALP (IU/L)	225.5(157–281)	107.5(90–136)	0.003
Symptoms			< 0.001
0	1 (6.3%)	12 (60%)	
1	9 (56.2%)	8 (40%)	
2	6 (37.5%)	0 (0%)	
Size (cm)	3.07 ± 1.32	2.85 ± 1.16	0.600
DR^*^	2.11 ± 0.60	2.71 ± 0.81	0.018
Location			0.053
Right	9 (56.3%)	9 (45%)	
Left	7 (43.7%)	11 (55%)	
Shape			< 0.001
Oval/round	1 (6.3%)	16 (80.0%)	
Irregular	15 (93.7%)	4 (20.0%)	
Echogenicity			0.009
Homogeneous	1 (6.3%)	10 (50%)	
Heterogeneous	15 (93.7%)	10 (50%)	
Calcification	3 (18.8%)	0 (0%)	0.078
Cystic change	9 (56.3%)	11 (55%)	0.650
Infiltration	13 (81.3%)	6 (30.0%)	0.002

DR^*^: the ratio between the maximum size and the minimum size of parathyroid lesions in longitudinal and transverse dimensions

The sonographic features are shown in Table 1 as well. As expected, size (the longest lesion diameter) was comparable between the two groups(*p* = 0.600). A significant difference in the tumor DR was found between the groups, with the PC/APT group having a lower DR value (*p* = 0.018). The location of the tumor was not different between the two groups (*p* = 0.053). The irregular shape was more prevalent in the PC/APT group than in the PA group (*p* <  0.001). Concerning echogenicity, heterogeneous mass was more frequent than homogeneous nodules in the PC/APT group (*p* = 0.009). The infiltrative border was highly prevalent in parathyroid carcinoma and atypical parathyroid tumor (*p* = 0.002). Intra-lesion cystic change was present in 56.3% (*n* = 9) of PC/APT group and 30% (*n* = 6) of PA group, showing no significant group differences (*p* = 0.650). The B-mode ultrasound and shear wave elastography findings of atypical parathyroid tumors and parathyroid carcinomas were also summarized in the Table 2. No significant differences were identified between the two entities with respect to the potentially risky factors indicated in the Tables 1 and 3, which render it more reasonable to classify them into the same group.

**Table 2:** The B-mode ultrasound and shear wave elastography findings of atypical parathyroid tumors and parathyroid carcinomas in our cohort

	DR^*^	Irregular shape	Infiltration	Heterogeneous Echogenicity	Calcification	Cystic change	Min SWV^a^/ Young’s modulus^b^	Mean SWV/ Young’s modulus	Max SWV/ Young’s modulus
Atypical parathyroid tumors (*n* = 8)	2.02	8/8(100%)	5/8(62.5%)	8/8(100%)	3/8(37.5%)	4/8(50%)	1.45/6.2	2.35/18.7	3.0/27.2
Parathyroid carcinomas(*n* = 8)	2.19	7/8(87.5%)	8/8(100%)	7/8(87.5%)	0/8(0%)	2/8(25%)	1.45/6.1	2.5/20.7	3.45//35.4
*p*-value	0.753	1.0	0.2	1.0	0.2	0.608	0.574/0.645	0.195/0.234	0.505/0.505

DR^*^: the ratio between the maximum size and the minimum size of parathyroid lesions in longitudinal and transverse dimensions; SWV^a^: the median value of the shear wave velocity(m/s); Young’s modulus^b^: the median value of the Young’s modulus (kPa)

**Table 3:** Areas under curve, best cut off points and diagnostic performance of quantitative and qualitative shear wave elastography features in differentiating between the parathyroid carcinoma/ atypical parathyroid tumor and parathyroid adenoma

SWE parameters^a^	Parathyroid carcinoma /atypical parathyroid tumor (*n* = 16)	Parathyroid adenoma (*n* = 20)	AUC(95%CI)	Cut-off	Sen	Spe	PPV	NPV	*p*-value
Min SWV(m/s)	1.45	0.75	0.644(0.459–0.829)	1.15	0.69	0.60	0.58	0.71	0.113
Mean SWV(m/s)	2.4	1.9	0.813(0.669–0.956)	2.35	0.56	0.95	0.90	0.73	< 0.001
Max SWV(m/s)	3.1	2.6	0.795(0.645–0.946)	2.70	0.75	0.75	0.71	0.79	0.006
Min modulus (kPa)	6.1	1.7	0.620(0.428–0.813)	3.9	0.69	0.60	0.58	0.71	0.105
Mean modulus (kPa)	18.2	11.1	0.852(0.720–0.983)	17.05	0.63	1.0	1.0	0.77	< 0.001
Max modulus (kPa)	28.45	20.35	0.794(0.639–0.948)	21.90	0.81	0.75	0.72	0.83	0.009
**Elastogram pattern**									< 0.001
Negative	3 (18.8%)	18(90%)							< 0.001
Void center	1 (6.2%)	2(10%)							> 0.05
Stiff rim	5 (31.2%)	0							< 0.001
Colored lesion	7 (43.8%)	0							< 0.001

*SWE* Shear wave elastography; ^a^: all the parameters were shown as the median values in the two group; *SWV* Shear wave velocity, *modulus* Young’s modulus, *Sen* Sensitivity, *Spe* Specificity, *PPV* Positive predictive value, *NPV* Negative predictive value

**Fig. 6:**
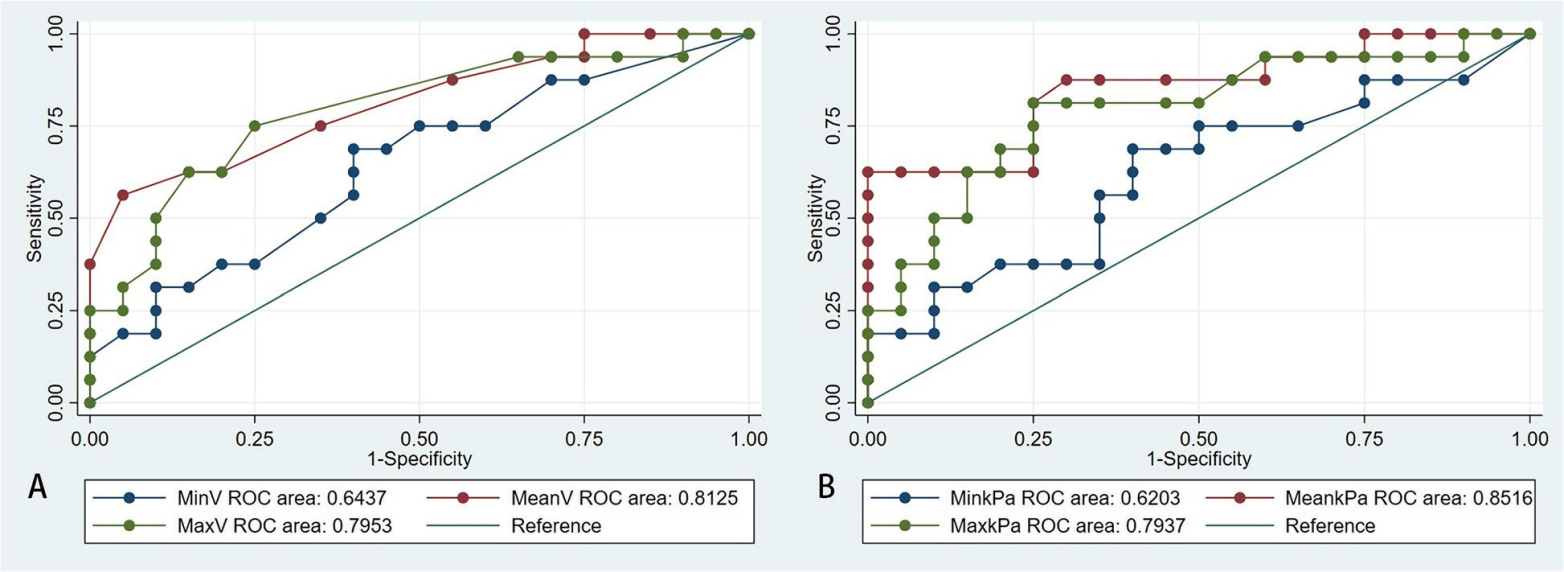
ROC curve analysis of different shear wave elastography parameters. **A** The area under the curve (AUC) of minimum, mean and maximum shear wave velocity (m/s) were 0.6437, 0.8125 and 0.7953, respectively. **B** The area under the curve (AUC) of minimum, mean and maximum Young’s modulus (kPa) were 0.6203, 0.8516 and 0.7937, respectively

The AUC for mean SWV, max SWV, mean Young’s modulus and max Young’s modulus were statistically significant (AUC = 0.813, *p* <  0.001; AUC = 0.795, *p* = 0.006; AUC = 0.852,p <  0.001; AUC = 0.794, *p* = 0.009; respectively). The min SWV and Young’s modulus were not useful in diagnosing PC/APT. No difference was observed between mean SWV/Young’s modulus and max SWV/Young’s modulus in recognizing PC/APT(*p* = 0.8582). The best cut-off value of mean SWV in predicting PC/APT was 2.35 m/s. The mean Young’s modulus showed an equally diagnostic prospect with the best cut-off value of 17.05 kPa. Meanwhile, the best cut-off values for max SWV and max Young’s modulus were 2.70 m/s and 21.90 kPa, respectively. For the qualitative SWE pattern classification, the negative pattern was predominant in the parathyroid adenoma group(*p* <  0.001), while the “rim of stiffness” and “colored lesion” pattern were representative in the parathyroid carcinoma/atypical parathyroid tumor group(p <  0.001,p <  0.001; respectively) (Table 3 and Fig. 6).

No correlations were identified between the lesion size and mean SWV, mean Young’s modulus, max SWV, max Young’s modulus (rs = 0.126, *p* = 0.462; rs = 0.114, *p* = 0.508; rs = 0.126, *p* = 0.463; rs = 0.168, *p* = 0.328; respectively). Mean SWV was not associated with iPTH (rs = 0.208, *p* = 0.224), but Max SWV and Young’s modulus slightly correlate with iPTH (rs = 0.398, *p* = 0.016; rs = 0.396, *p* = 0.017; respectively).

## Discussion

Parathyroid carcinoma and atypical parathyroid tumors remain a conundrum to diagnose preoperatively. Lacking efficient methods to identify them before surgery could result in insufficient treatment. In our prospective study, the SWE characteristics of the two entities were described and the differences between them and parathyroid adenomas were demonstrated by values of SWE and the elasticity map. To our knowledge, this is the first study trying to differentiate parathyroid tumors with risk of recurrence from parathyroid adenomas with favorable prognosis using the 2D-SWE method.

We classified parathyroid carcinoma and atypical parathyroid tumor into a group in this study due to the commonly equivocal diagnosis of the two entities except for a long-term follow-up [5]. Meanwhile, the 2022 WHO classification of parathyroid tumors changed the nomenclature of “atypical parathyroid adenoma” into “atypical parathyroid tumor”, which reflected a parathyroid neoplasm of uncertain malignant potential [6]. Along with worrisome clinical and biochemical features, atypical parathyroid tumors could harbor histology aberrances often seen in parathyroid carcinoma including band-like fibrosis, adherence to adjacent structures, and Ki-67 labeling index > 5%. Compared with the malignant lesions with a high risk of recurrence, atypical parathyroid tumors with parafibromin deficiency have a low risk of recurrence and subsequent CDC73 gene sequencing is necessary for this entity [7]. Therefore, it is not only difficult but meaningless to distinguish cancer and atypical tumor before surgery. Preoperative diagnosis of both parathyroid tumors together would be appropriate to guide the choice of surgical approach.

In recent decades, elastographic techniques have experienced rapid developments with device iterations. From strain elastography to acoustic radiation force impulse (ARFI) [8–10], from point shear wave elastography to 2D shear wave elastography [11–13], previous studies employed these emerging methods to differentiate parathyroid adenoma from parathyroid hyperplasia, reactive lymph nodes or thyroid nodules. Given these researches, some scholars suggested minimally invasive parathyroidectomy for those single parathyroid lesions with high SWV because they are more likely to be parathyroid adenomas rather than parathyroid hyperplasia. However, they have ignored an important clinical scenario that the parathyroid carcinoma and atypical parathyroid tumors could be identified occasionally and they are indeed stiffer than parathyroid adenomas according to our study. Tumor stiffness is proved to correspond with tumor progression and invasiveness in cancers of different origins. The thyroid and breast malignancies have shown greater stiffness than their benign counterparts in many studies [14, 15]. Under such a situation, we firstly reported the cut-off value of 2.35 m/s in SWV and 17.05 kPa in Young’s modulus suggestive of the parathyroid carcinoma and atypical parathyroid tumor using 2D-SWE. Additionally, 2D-SWE has overcome the main limitations of previous studies utilizing operator-dependent strain elastography or small lesion-unfriendly VTQ technique, which may enhance the practicability in the clinical setting [16].

Four studies using the same Supersonic Imagine Aixplorer System to evaluate the parathyroid lesions were identified in the PubMed database. In a study carried out by Golu et al., the authors found that the mean elasticity indexes of parathyroid lesions were 10.2 kPa, which is comparable to our measurement of 10.89 kPa for parathyroid adenoma [17]. Stangierski et al. reported that the mean elasticity of parathyroid adenoma (5.2 ± 7.2 kPa) was significantly lower than benign thyroid nodules(24.3 ± 33.8 kPa), and they suggested that the negative predictive value of low elasticity was high enough to exclude suspicion of parathyroid adenomas [18]. Accordingly, we found parathyroid adenomas were more elastic than their counterparts, and the mean Young’s modulus > 17.05 kPa was reliable to diagnose suspicious parathyroid malignancies with excellent specificity. Amzar et al. found that the cut-off values of mean SWE confirmed for parathyroid adenoma and parathyroid tissue were 5.96 kPa and 9.58 kPa, respectively [19]. The elasticity of parathyroid tissue (parathyroid lesions in primary and secondary hyperparathyroidism) was significantly lower than thyroid or muscle tissue in their research. Cotoi et al. reported a cut-off value below 7 kPa to diagnose the parathyroid adenoma, and color maps in strain elastography rather than strain ratios were useful in identifying parathyroid adenomas [20]. In our study, parathyroid carcinoma and atypical parathyroid tumor showed significantly larger stiffness than parathyroid adenoma given the mean and max SWE velocities as well as the corresponding Young’s modulus. The mean Young’s modulus and shear wave velocity demonstrated the best diagnostic efficacy based on the area under the ROC curve (0.813, 0.852, respectively). Nevertheless, for achieving a balanced diagnostic performance, the max Young’s modulus was advised to differentiate the two group lesions with a sensitivity of 0.81 and specificity of 0.75. Moreover, the correlation between the max SWV/Young’s modulus and PTH may be explained by the ultrasound echogenicity features investigated by Li et al. [21] The authors reported that the median serum PTH level of the hypoechoic parathyroid lesions was higher than those of the iso-hyperechogenic group. They ascribed the finding to the histopathologic components in parathyroid tumors. The iso-hyperechogenic areas mainly correspond to non-functioning lipocytes, loose edema, connective tissues, or normal parathyroid tissues by pathology, which are usually soft tissues with relatively low SWE measuring values. In contrast, the PC and APT are mostly full of actively proliferated chief cells and fibrous bands whose elasticities are commonly high [22]. The max SWV/Young’s modulus could embody the histopathologic structures that are strongly associated with a high PTH level.

The elastogram of a parathyroid lesion reflects the tumor stiffness more comprehensively by containing the minimum, mean and maximum value of SWE information all together in a color-coded image. It is expected that the negative pattern was more common in the parathyroid adenoma group, which may due to the homogeneous and soft nature of parathyroid adenomas. The components of this benign parathyroid neoplasm are relatively simple, with high tumor cellularity, low amounts of connective tissue, fibrosis and necrosis. The “Stiff rim” sign was emphasized by Zhou et al. in differentiating between benign and malignant breast lesions [23]. The mechanism behind this qualitative SWE feature may lie in the infiltration of cancer cells into the surrounding tissues. “Colored lesion” on elastogram often indicates the increased stiffness around and within the targeted lesions. BE1 multinational study had found that this sign could improve the diagnostic performance by downgrading or upgrading breast lesions classified into 4a or 3 grades [24]. Accordingly, the pathologic diagnosis of PC should include the unequivocal infiltration of adjacent structures, and vascular or neural tissues, while the atypical parathyroid tumors are invariably characterized by thickened connective tissues, adherence to adjacent structures, or band-like fibrosis [25]. These pathological features are closely associated with our findings of prevailed stiff rim and colored lesion patterns in the PC/APT group. The signal void pattern did not show diagnostic value in this study. This phenomenon often derives from liquid formation or extremely rigid areas inside the tumor that hinders shear wave propagation. The liquid formation could be cystic degeneration, necrosis, or bleeding in pathology and is often displayed as anechoic areas on ultrasonography [26]. According to our previous study, these changes are associated with the size of parathyroid tumors and do not show a difference between parathyroid carcinoma and parathyroid adenoma/hyperplasia [27]. The extremely rigid areas usually found in malignant tumors are confirmed as the presence of intralesional dense collagen deposits in previous breast cancer research [28]. Although similar histopathological features are also reported in PC/APT, the signal void pattern was only observed once in our cohort. We assume that, in most cases, the collagen deposits and fibrosis in PC/APT are not so dense as to cause an extremely rigid area that invalid the SWE analysis.

This study has some limitations. Firstly, the small scale of cases restricts further statistical analysis for exploring independent risk factors of SWE parameters. The improved diagnostic performance of adding SWE to conventional US and clinical features could not be estimated too. Secondly, quantitative values of SWE are believed not possible to extrapolate between two different devices. Thirdly, the interobserver variability in the evaluation of parathyroid lesions using 2D-SWE was not assessed, which requires further research.

## Conclusions

Atypical parathyroid tumor or parathyroid carcinoma is often observed in colored and stiff rim pattern on elastogram and showing the mean SWE value > 2.35 m/s or the mean Young’s modulus > 17.05 kPa, whereas a negative elastogram with a less SWE value is characteristic of parathyroid adenoma. The mean SWV and Young’s modulus are good at recognizing the parathyroid adenoma with excellent specificities, while the max SWV and Young’s modulus are more sensitive to distinguish the PC and APT compared with other parameters.

## Data Availability

The datasets generated and/or analyzed during the current study are available from the corresponding author upon reasonable request.

## Abbreviations

PC: Parathyroid carcinoma
APT: Atypical parathyroid tumor
PA: Parathyroid adenoma
PHPT: Primary hyperparathyroidism
SWE: Shear wave elastography
SWV: Shear wave velocity
US: Ultrasonography
DR: Diameter’s ratio
ROI: Region of interest
PTH: Parathyroid hormone
ARFI: Acoustic radiation force impulse
